# A Lightweight RFID Mutual Authentication Protocol Based on Physical Unclonable Function

**DOI:** 10.3390/s18030760

**Published:** 2018-03-02

**Authors:** He Xu, Jie Ding, Peng Li, Feng Zhu, Ruchuan Wang

**Affiliations:** 1 School of Computer Science, Nanjing University of Posts and Telecommunications, Nanjing 210023, China; 1016041216@njupt.edu.cn (J.D.); lipeng@njupt.edu.cn (P.L.); zhufeng@njupt.edu.cn (F.Z.); wangrc@njupt.edu.cn (R.W.); 2 Jiangsu High Technology Research Key Laboratory for Wireless Sensor Networks, Nanjing 210003, China

**Keywords:** RFID technology, Physical Unclonable Function, lightweight cryptography, mutual verification

## Abstract

With the fast development of the Internet of Things, Radio Frequency Identification (RFID) has been widely applied into many areas. Nevertheless, security problems of the RFID technology are also gradually exposed, when it provides life convenience. In particular, the appearance of a large number of fake and counterfeit goods has caused massive loss for both producers and customers, for which the clone tag is a serious security threat. If attackers acquire the complete information of a tag, they can then obtain the unique identifier of the tag by some technological means. In general, because there is no extra identifier of a tag, it is difficult to distinguish an original tag and its clone one. Once the legal tag data is obtained, attackers can be able to clone this tag. Therefore, this paper shows an efficient RFID mutual verification protocol. This protocol is based on the Physical Unclonable Function (PUF) and the lightweight cryptography to achieve efficient verification of a single tag. The protocol includes three process: tag recognition, mutual verification and update. The tag recognition is that the reader recognizes the tag; mutual verification is that the reader and tag mutually verify the authenticity of each other; update is supposed to maintain the latest secret key for the following verification. Analysis results show that this protocol has a good balance between performance and security.

## 1. Introduction

Radio frequency identification technology is able to recognize objects and people automatically, and it can also automatically obtain related data of recognized objects, which is the non-contract recognition technique [1]. Because of this, the recognition of radio frequency identification (RFID) does not only need the artificial interference, but also work well in the severe environment. Recently, most RFID systems are based on the electric induction [2]. Attaching a RFID tag on an object, which involves the information of this object, the dedicated recognition terminal can recognize this attached object through reading the tag. In addition, since RFID products read data does not require light source and can pass through external material, and the service life is durable, compared with bar code, RFID products have more advantages [3].

By now, RFID has been used into many areas, such as supply chain management, electric passport, credit card, driving license [4,5], vehicle system (charging system, keyless entry systems), entrance guard card (building gate, public transport) and health care. Especially, in the USA, Japan, and other developed countries, they have equipped with advanced and mature RFID systems [6]. Some retailers have invested RFID technology, and also authorized RFID producers to attach tag on their goods, so that the low-budget RFID tags are pervasively produced. Wal-Mart passed a resolution, which producers must sufficiently take advantage of the RFID, attaching RFID tags on all products to reduce manpower and material resources [7].

**Figure 1:**
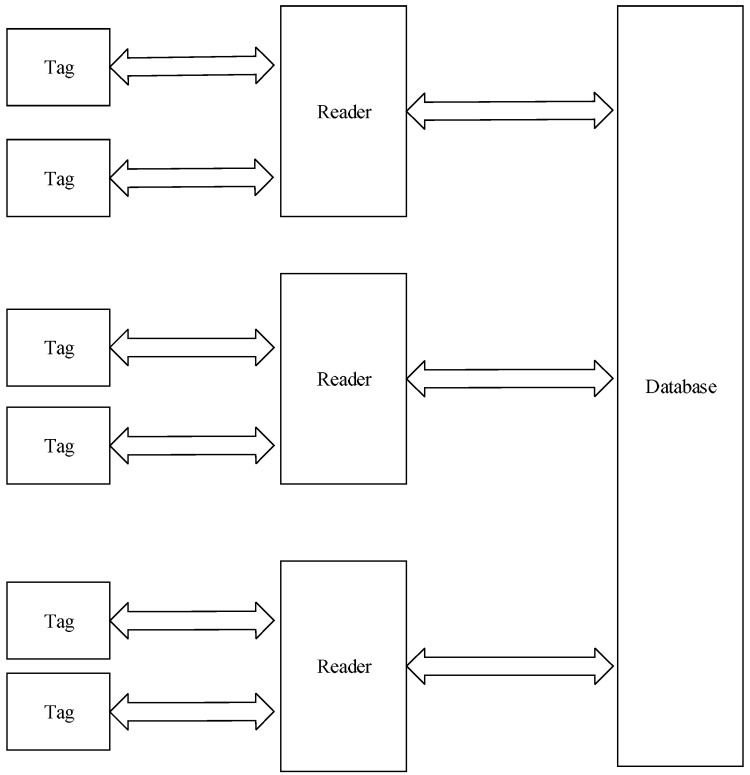
Radio frequency identification (RFID) system framework.

Generally, a typical RFID framework is composed of a reader, tag and a database [8], which is shown in Figure 1.

(1) Reader: The main function is to transfer energy to the tag via radio frequency and read data of tag or write data to tag [9]. In common RFID systems, reader still needs to exchange data with database. A reader is composed of the oscillating circuit, communication channel, and controller.
(2) Tag: According to the self-contained power or not, tag is classified into active tag, semi-passive tag and passive tag; based on the frequency, tag is classified into low-frequency tag, high-frequency tag and ultrahigh frequency tag [10]. By various applications, the proper tags are needed to be chosen.
(3) Database: It stores all information of tags which indicate all objects.

Mechanism of RFID systems: Firstly, reader sends signals via antenna, and tag receives signal and sends internal tag data. Then, reader receives and verifies the tag data. Finally, reader sends verification result to the host computer which is connected to a database.

Because RFID has the advantages of supporting dynamic real-time communication, fast recognition, easy to read, low cost, it is widely applied into various areas. Nevertheless, when RFID brings convenience, at the same time, its security problem is gradually exposed. In detail, during a simple write and read process, especially for the read or write on a passive tag, the read or write operation will happen when tag is close to a reader with the information exchange. Later, with the existence of a large number of fake and counterfeit RFID products, although researchers continuously find anti-fake measures, it is unable to completely eradicate them. Fake and counterfeit products make massive loss for world politics, economy, and culture, it has been a worldwide problem [11]. Clone tag is a kind of serious security threat for RFID systems. If attackers acquire the complete information of a tag, they could be able to obtain the unique identifier. In addition of this identifier, tag has no other identify, clone tag is therefore difficult to distinguish from the real one. In addition, as long as the legal tag data is obtained, attacker can launch such copy or clone attack.

In this case, researchers have taken measures to defend against clone attack, such as tag deactivation, encryption algorithms [12], identify verification [13] and hash code [14]. Reference [15] showed a mutual RFID verification protocol by using the elliptic curve cryptograph (ECC), which depends on ECC to enhance the ability of anti-clone. In the literature [16], authors gave a tag encryption algorithm without storage of the secret key. Instead, it depends on the hash value stored in the host computer. Reference [17] gave another RFID verification protocol based on hash, which provides already modified identifier to increase the privacy of verification. These mentioned protocols above rely on the security code which involve tag and the host computer, as well as one-way hash function.

Since the small and limited storage space in the tag, those solutions are based on the complex encryption algorithm which is similar to the hash function and it cannot be applied into the low-cost tag [18]. In such low-cost RFID systems, the biggest challenge to guarantee the security and privacy comes from the sparse tag resource [19]. To solve this problem, some lightweight encryption algorithms without hash function are put forward, and the method to identify clone tag as well [20,21]. Lehtonen raised a method based on statistics to detect clone tag from the RFID track [22]. Zannetti showed a method to detect clone attack with the protection of privacy [23].

**Figure 2:**
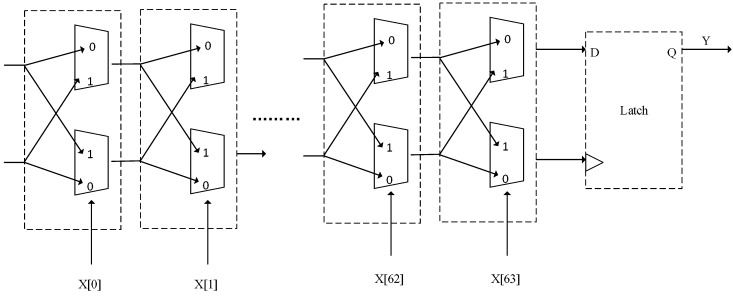
PUF subgrade circuit diagram.

Another anti-clone tag method is proposed by using the PUF technique. PUF technique is a breakthrough of the semi-conductor security techniques. PUF is a “biometric” identification technique in chips, which is also called the “chip DNA” technique [24]. PUF obtains the unique secrets from each chip. This secret information can be used to verify the authenticity of a chip, in the safe-guarding and anti-counterfeiting area, PUF has great application perspective. In the production of each chip, the tiny difference among chips unavoidably exist, although the chip design and production are the same, it is impossible to produce two chips which are completely same, so that PUF techniques can enhance anti-clone function [25]. The PUF judge circuit is shown as Figure 2 [26]. In Figure 2, the circuit is a decision-based PUF delay circuit. After inputting X[0], the circuit generates two delay paths whose length are equal, and the input signal is sent to the two paths at the same time. The decision module will choose the path that the input signal first reaches the destination. If the signal which is connected to the D point arrives firstly, it will output 1, otherwise it will output 0. Different physical manufacturing processes result in different outcomes which will give rather different outputs to the same input. PUF circuit receives a search command as an input signal and produces a unique serial feedback, which is verified by command/response mechanism. Different PUF circuits have various delay features, so that their transport speed is different and time of two signals passing PUF is different. The tail of a PUF circuit is an arbiter, and is able to judge the statue of signals before and after to output “1” or “0”. A same signal passing two different PUF circuits can produce different outputs, and because that input signal determines the transport rout in PUF circuit, the different input signals are corresponding to different outputs.

In 2010, Kulseng proposed a lightweight mutual verification protocol based on PUF [27]. This lightweight protocol uses the PUF and linear feedback shift register (LFSR), rather than uses the complex plus and minus operations, which is very suitable for low-cost RFID tag, but it indicates the security problems of lightweight mutual verification [28].

This paper is structured as follows: section I introduces related research results about RFID security. Section 2 describes Kulseng’s lightweight mutual verification protocol and the shortcomings of this protocol. Section 3 introduces our lightweight mutual verification protocol. Section 4 uses GNY logic analysis to prove the availability of our protocol. Section 5 executes the security analysis on our protocol. Section 6 simulates our lightweight mutual verification protocol and evaluate it in the experiments. Section 7 gives the performance analysis. At last, we conclude the paper.

## 2. Kulseng’s Mutual Verification Protocol and Its Security Analysis

This section specifically analyses the Kulseng’s mutual verification protocol based on the PUF. In this paper, it is called K protocol in the following. Some definitions of K protocol are shown in Table 1.

### 2.1. Protocol Process

**Figure 3:**
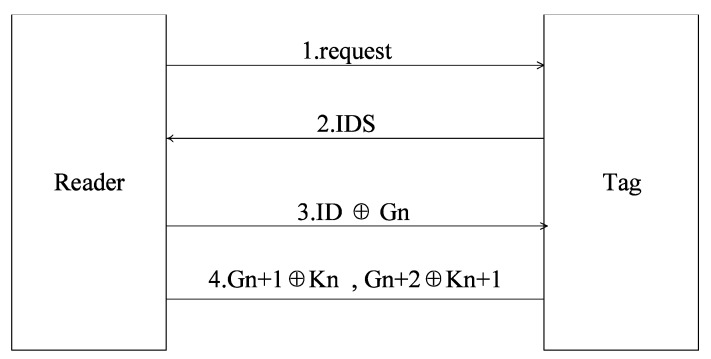
Kulseng’s mutual verification protocol.

Figure 3 shows the Kulseng’s mutual verification protocol. The specific steps are listed in the following.

S1: Reader firstly sends a search request to the tag.

S2: After receiving the request, the tag sends the IDS to reader.

S3: After receiving the IDS from the tag, reader searches this IDS in database. If this IDS can be found, it means that the database stores this IDS, and the tag is successfully recognized and reader sends $\mathrm{ID}\;⊕{\;G}_{n}$ to the tag. If not, this tag can not be recognised and the protocol stops.

S4: After receiving $\mathrm{ID}\;⊕{\;G}_{n},\;$by calculating $\mathrm{ID}\;⊕{\;G}_{n}\;⊕\;\mathrm{ID}\;$to obtain ${\;G}_{n}$ of $\mathrm{ID}\;⊕{\;G}_{n}$ sent from reader, and tag compares this with its own${\;G}_{n}$. If they are different, it means that the reader is not been verified, and the protocol stops. Otherwise, this reader is verified. The tag executes following operations:

① Calculating following value:
  $$G_{n+1}=\mathrm{PUF}\left({\;G}_{n}\right),{\;G}_{n+2}=\mathrm{PUF}\left({\;G}_{n+1}\right),{\;K}_{n}=F\left({\;G}_{n}\right),{\;K}_{n+1}=F\left({\;K}_{n}\right)$$
② Calculating the following value and sending them to the reader.
  $${\;K}_{n}⊕{\;G}_{n+1},{\;K}_{n+1}⊕{\;G}_{n+2}$$
③ Update tag data:
  $$\mathrm{IDS}=F\left(\mathrm{IDS}\;⊕{\;G}_{n}\right),{\;G}_{n}=G_{n+1}$$

S5: After receiving $K_{n}⊕{\;G}_{n+1},{\;K}_{n+1}⊕{\;G}_{n+2}$, reader judges whether ${\;K}_{n}⊕{\;G}_{n+1}$ = $F\left({\;G}_{n}\right)\;⊕{\;G}_{n+1}$ or not. If not, this tag is not verified, and the K protocol stops; if it is equal, tag is verified and reader executes the following operations:

① Calculating value of ${\;G}_{n+2}$:
  $${\;K}_{n+1}⊕{\;G}_{n+2}\;⊕\;F\left(F\left({\;G}_{n}\right)\right)$$
② Updating reader data by the calculated value of ${\;G}_{n+2}$:
  $$\mathrm{IDS}=F\left(\mathrm{IDS}\;⊕{\;G}_{n}\right),{\;G}_{n}=G_{n+1},{\;G}_{n+1}=G_{n+2}$$

### 2.2. Security Analysis of K protocol

#### 2.2.1. Data Confidentiality

Attacker firstly intercepts a complete verification process to obtain $\mathrm{ID}\;⊕{\;G}_{n},{\;K}_{n}⊕{\;G}_{n+1}{\;\mathrm{and}\;K}_{n+1}⊕{\;G}_{n+2}.\;$Tag updates its data after a complete verification, and $\mathrm{IDS}=F\left(\mathrm{IDS}\;⊕{\;G}_{n}\right)\;$is updated at the same time. In the next verification, attacker pretends to be a reader and sends a request to tag, and the tag sends its IDS to attacker after receiving the request from attacker, in which the IDS = F$\left(\mathrm{IDS}\;⊕{\;G}_{n}\right).\;$The attacker has four values: $\mathrm{ID}\;⊕{\;G}_{n},{\;K}_{n}⊕{\;G}_{n+1},{\;K}_{n+1}⊕{\;G}_{n+2}$ and $\left(\mathrm{IDS}\;⊕{\;G}_{n}\right)$. Here, F is the function representing Linear Feedback Shifting Register (LFSR). According to the LFSR, if the attacker knows the LFSR’s characteristic polynomial and output, the attacker can know the seed of LFSR via the matrix multiplication. In this way, combining the IDS in last verification and $F\left(\mathrm{IDS}\;⊕{\;G}_{n}\right)$ obtained at this time, attacker can easily know the secret key ${\;G}_{n}$ used in the last verification, and use XOR between${\;G}_{n}\;\mathrm{and}\;\mathrm{ID}\;⊕{\;G}_{n},\;$then attacker can know the ID of tag.

#### 2.2.2. Desynchronized Attack

Attack one: Intercept the last information sent by tag to reader $({\;K}_{n}⊕{\;G}_{n+1},{\;K}_{n+1}⊕{\;G}_{n+2}$).

Assume attacker is intercepting a verification process, after the tag confirms the reader, tag updates its data and sends ${\;K}_{n}⊕{\;G}_{n+1},{\;K}_{n+1}⊕{\;G}_{n+2\;}$ to the reader. At this moment, the attacker can obtain this information without being received by the reader. Because reader cannot verify the tag, the database cannot update the corresponding secret key value to the tag, which causes the data of the tag and the database are not synchronized. In the following verification, due to the difference between ${\;G}_{n}$ of the tag and ${\;G}_{n}$ of the datab ${\;K}_{n}$ ase, tag would refuse service.

Attack two: Modify ${\;K}_{n+1}⊕{\;G}_{n+2}$ of $(⊕{\;G}_{n+1},{\;K}_{n+1}⊕{\;G}_{n+2}$) sent from tag to reader.

Assume an attacker is intercepting a verification process, after the tag confirms the reader, tag updates its own data and sends ${\;K}_{n}⊕{\;G}_{n+1},{\;K}_{n+1}⊕{\;G}_{n+2}$ to the reader. Then, attacker modifies ${\;K}_{n+1}⊕{\;G}_{n+2}$ of the information sent to the reader, such as modifying into (${\;K}_{n+1}⊕{\;G}_{n+2}\;⊕{\;r}_{1}$), which is finally sent to the reader. After reader receives${\;K}_{n}⊕{\;G}_{n+1},{\;K}_{n+1}⊕{\;G}_{n+2}\;⊕{\;r}_{1}$, reader judges whether ${\;K}_{n}⊕{\;G}_{n+1}$ = $F\left({\;G}_{n}\right)\;⊕{\;G}_{n+1}$ or not to verify the tag. Because the attacker does not modify${\;K}_{n}⊕{\;G}_{n+1}$, reader successfully verifies the tag. After this, reader uses ${\;K}_{n+1}⊕{\;G}_{n+2}\;⊕{\;r}_{1}\;$to execute the following operations as usual.

① Calculating${\;G}_{n+2}$:
  $${\;K}_{n+1}⊕{\;G}_{n+2}\;⊕{\;r}_{1}⊕\;F\left(F\left({\;G}_{n}\right)\right)$$
  Now,${\;G}_{n+2}$ is updated by${\;G}_{n+2}=G_{n+2}\;⊕{\;r}_{1}$.
② Using${\;G}_{n+2}$ to update reader information:
  $$\mathrm{IDS}=F\left(\mathrm{IDS}\;⊕{\;G}_{n}\right),{\;G}_{n}=G_{n+1},{\;G}_{n+1}=G_{n+2}⊕{\;r}_{1}$$

This causes the desynchronization between tag data and database. In the following verification, due to the difference between ${\;G}_{n+1}$ of the database and ${\;G}_{n+1\;}$of the tag, verification for the tag is failed.

## 3. Proposed Protocol

Due to the shortcoming of Kulseng’s lightweight mutual verification protocol that is based on the PUF, we will introduce our proposed protocol in this section. Some definitions of proposed protocol are shown in Table 1 at the previous section.

**Figure 4:**
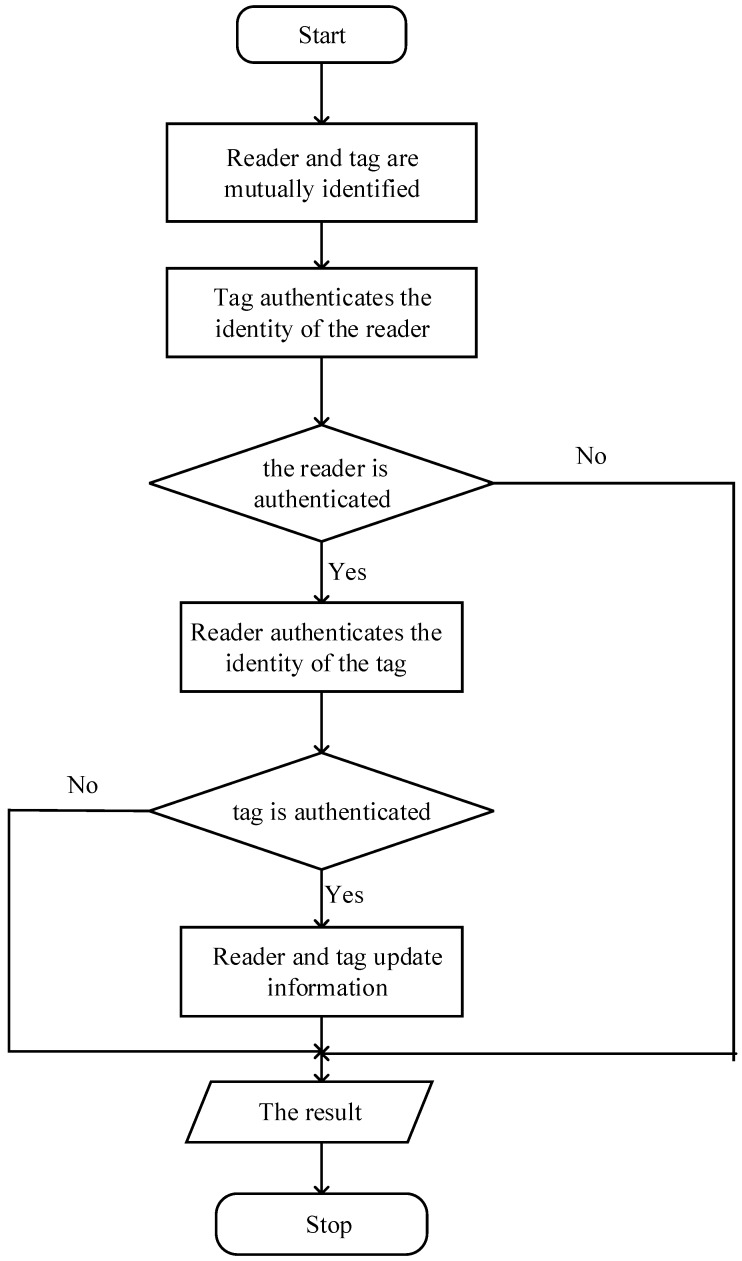
Single tag certification process.

In this protocol, RFID tag is embedded with the PUF module, which makes each RFID tag to produce a unique secret key based on its own circuit, so as to defend against the clone attack. Initially, each RFID tag stores three values: ①${\;P}_{n}=\mathrm{PUF}\left(\mathrm{challenge}\right)$, ②FID and ③${\;K}_{n}$. The first value is the dedicated secret value of the tag; the second value is the IDS used during the mutual verification between the tag and the host computer; the third one is the shared secret key by the reader and the tag. After each verification, the secret key $P_{n}$, shared secret key${\;K}_{n}\;$and FID would be updated. For each tag, the database stores two set of values: $\left\{{\mathrm{FID}}^{\mathrm{old}},{\;P}_{n}^{\mathrm{old}},{\;P}_{n+1}^{\mathrm{old}},\;\mathrm{ID},{\;K}_{n}^{\mathrm{old}}\right\}$ and $\left\{{\mathrm{FID}}^{\mathrm{new}},P_{n}^{\mathrm{new}},P_{n+1}^{\mathrm{new}},K_{n}^{\mathrm{new}}\right\}$. The later value set is the current tag value, and the prior value set is the tag value of the last communication. This verification protocol contains three parts: tag recognition, mutual verification and update. Tag recognition is to verify the tag’s authenticity; mutual verification is to use the verified tag to verify reader’s authenticity; update is to store the latest secret key for the following verification. Figure 4 shows the certification process of proposed protocol.

In this paper, the backstage database cannot meet the requirements because it should store all the tag information resulting in a very large amount of data. In order to solve the above problems, the HBase of big data technology is used to store the data. HBase which is a distributed and column-oriented is an open source database. A table in HBase can have hundreds of millions of rows and millions of columns, and the data in each unit can have multiple versions for backup.

### 3.1. Tag Verification

In the tag verification process, reader firstly sends a search request to tag, after receiving the request, tag produces a random number${\;r}_{1}$, and sends $r_{1}$ with FID to the reader. After receiving the random number $r_{1}$ and FID, reader uses FID to search data in the host database. If $\mathrm{FID}={\mathrm{FID}}^{\mathrm{new}},\;$it means that the database contains this FID, and this tag can be verified without being attacked previously. In addition, the reader uses $\left\{{\mathrm{FID}}^{\mathrm{new}},{\;P}_{n}^{\mathrm{new}},{\;P}_{n+1}^{\mathrm{new}},{\;K}_{n}^{\mathrm{new}}\right\}$ to execute mutual verification with the tag. However, in the previous verification, tag might be intercepted, resulting in the update of the database, while the tag data is not updated. In this case, reader uses $\left\{{\mathrm{FID}}^{\mathrm{old}},{\;P}_{n}^{\mathrm{old}},{\;P}_{n+1}^{\mathrm{old}},\;\mathrm{ID},{\;K}_{n}^{\mathrm{old}}\right\}$ to execute the mutual verification with this tag. If both of the new and old FID cannot match with the FID of the reader, this protocol stops.

### 3.2. Mutual Verification

The purposes of Mutual verification is to verify the identity of both tag and reader. Reader firstly produces a random number $r_{2}$, combining FID, secret key${\;P}_{n+1}$, shared key $K_{n}$ and random number $r_{1}$ according to the Equations (1) and (2) to calculate A and B. Then, reader sends A||B to the RFID tag.

(1) $$A=\mathrm{FID}\;⊕{\;P}_{n+1}⊕{\;K}_{n}\;⊕{\;r}_{1}⊕{\;r}_{2}$$

(2) $$B=\left(r_{1}<\;<\;<8\right)\;\&\;\left({\;r}_{2}<\;<\;<1\right)\;⊕\;\left({\;P}_{n+1}<\;<\;<2\right)$$

After receiving A||B, tag calculates D and E by Equations (3) and (4), Equation (5) deduces the random number $r_{2}^{\prime }$ of the reader and further uses $r_{2}^{\prime }$ to obtain$\;B^{\prime }\;$by Equation (6).

(3) $$D=\mathrm{PUF}\left(P_{n}\right)$$

(4) E=PUF(D)

(5) $$r_{2}^{\prime }=A\;⊕\;\mathrm{FID}\;⊕\;D\;⊕{\;K}_{n}⊕{\;r}_{1}$$

(6) $$\;B\;\prime =\left(r_{1}<\;<\;<8\right)\;\&\;\left(r_{2}^{\prime }<\;<\;<1\right)\;⊕\;\left(D<\;<\;<2\right)$$

Then, tag compares whether B and $B^{\prime }$ are equal or not. If not, the reader is fake, and verification terminates; otherwise, the reader passes the verification, and the protocol goes on for the next step. At the same time, with previously calculated D, E and deduced random number $r_{2}^{\prime }$, by Equation (7) F is calculated, which is also used to deduce the random number $r_{2}^{\prime }$, F and FID of the tag. By Equation (8). H is calculated and F||H is sent to reader.

(7) $$F=D\;⊕\;E⊕{\;r}_{2}^{\prime }$$

(8) $$H=\left(\mathrm{FID}<\;<\;<8\right)\;\&\;\left(F<\;<\;<1\right)\;⊕\;\left(r_{2}^{\prime }<\;<\;<2\right)$$

After receiving F||H, by Equation (9), reader deduces secret key $E^{\prime }$ of the tag, and this deduced key is used to obtain $E^{\prime }$ random number $r_{2}$ and secret key $P_{n+1}$. With Equation (10),  F ′ is calculated. Then, using the FID of this tag,  F ′, random number $r_{2}$, by Equation (11).${\;H}^{\prime }\;$is calculated.

(9) $$\;E\;\prime =F\;⊕\;r_{2}\;⊕\;{\;P}_{n+1}$$

(10) $$\;F\;\prime =\;E\;\prime \;⊕\;r_{2}\;⊕\;P_{n+1}$$

(11) $$\;H\;\prime =\left(\mathrm{FID}<\;<\;<8\right)\;\&\;\left(\;F\;\prime <\;<\;<1\right)\;⊕\;\left(r_{2}<\;<\;<2\right)$$

At this moment, reader compares whether H and  H ′ are equal or not. If it is equal, the tag has been verified before and the mutual verification terminates. If not, this tag is fake, and the verification stops.

### 3.3. Update Process

After verifying the tag, update starts. During this process, firstly, the host database updates, then the tag updates. The process of updating the database is classified into two cases. In the tag update, if the database uses $FID^{oid}$ and FID to match, the host database does not need to update; if the database uses $FID^{new}$ and FID of the tag to match, the database should be updated in following way:

$$P_{n}^{\mathrm{new}}=P_{n+1}^{\mathrm{new}},{\;P}_{n+1}^{\mathrm{new}}=\;E\prime$$

$${\;K}_{n}^{\mathrm{new}}=\left(K_{n}^{\mathrm{new}}<\;<\;<8\right)\;\&\;\left(r_{2}^{\prime }<\;<\;<1\right)\;⊕\;\left(r_{1}<\;<\;<2\right)$$

$${\mathrm{FID}}^{\mathrm{new}}=\left({\mathrm{FID}}^{\mathrm{new}}<\;<\;<8\right)\;\&\;\left(r_{2}^{\prime }<\;<\;<1\right)\;⊕\;\left(r_{1}<\;<\;<2\right)$$

$${\mathrm{FID}}^{\mathrm{old}}={\mathrm{FID}}^{\mathrm{new}},{\;P}_{n}^{\mathrm{old}}=P_{n}^{\mathrm{new}},{\;P}_{n+1}^{\mathrm{old}}=P_{n+1}^{\mathrm{new}},{\;K}_{n}^{\mathrm{old}}=K_{n}^{\mathrm{new}}$$

**Figure 5:**
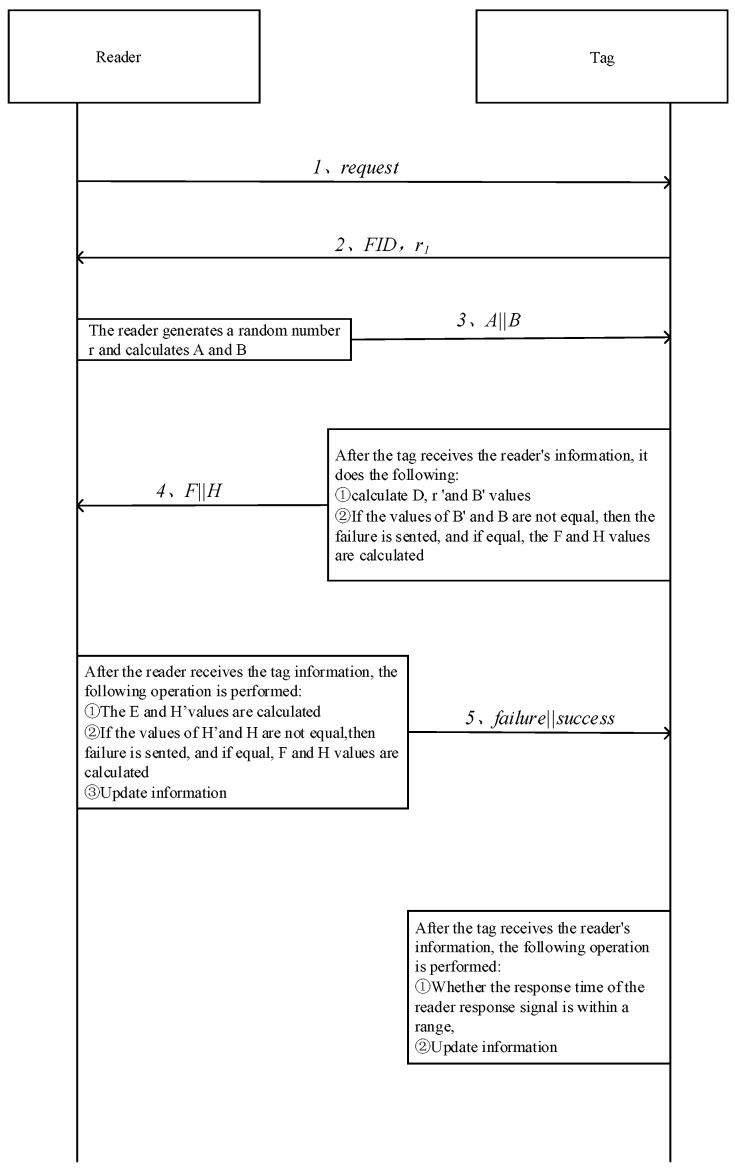
Time sequence diagram of single tag authentication.

If reader sends success command to the tag, and the processing time of the reader is within the acceptable range, the tag would be updated in the following ways, which is shown in Figure 5.

$$P_{n}=D$$

$$\mathrm{FID}=\left(\mathrm{FID}<\;<\;<8\right)\;\&\;\left(r_{2}^{\prime }<\;<\;<1\right)\;⊕\left(r_{1}<\;<\;<2\right)$$

$$K_{n}=\left(K_{n}<\;<\;<8\right)\;\&\;\left(r_{2}^{\prime }<\;<\;<1\right)\;⊕\;\left(r_{1}<\;<\;<2\right)$$

## 4. Proof of Security

In this section, we use GNY logic [29] to prove our protocol. The GNY logic was proposed by Gong, Needham and Yahalom in 1990. It is a logic that analyzes the authentication protocol. The GNY logic uses a completely different state search tool, which includes a collection of beliefs maintained by each subject and a collection of inference rules that get new beliefs from the old beliefs. BAN logic has a very simple, intuitive set of rules, so it is easy to use. As it is pointed out in the reference [29], GNY logic can be used to find serious errors in the protocol, which has attracted widely attention by security researchers. The application of GNY logic has epoch-making significance. It greatly promoted the development of formal verification of security protocols, and inspired many methods of formal verification of security protocols.

### 4.1. GNY Logic Expression

A|≡B: A trusts message B.

A|∇B: A receives message B.

A|~B: A sends message B.

A∋B: A has message B.

(B, C): connecting message B and message C.

#(B): message B is the latest, which means that message B has never been sent before.

{B}_k_: message B is encrypted by secret key k.

$A\overset{k}{↔}D$: A and D share secret key k to communicate, which means only A and D or the trusted third party know the secret key k.

### 4.2. Principle of the GNY Deduction

Principle 1: $\frac{A|\equiv \#\left(B\right)}{A\left|\equiv \#\left(B,C\right),A\right|\equiv \#\left(F\left(B\right)\right)}$

Principle 1 indicates: if A trusts that message B is the latest, then it can be deduced that A also trusts the connection between B and C is the latest, and the mapping is trusted as the latest by A whose image is B.

Principle 2: $\frac{A∋B,A∋C}{A∋\left(B,C\right),A∋\left(F\left(B,C\right)\right)}$

Principle 2 indicates: If A has message B and C, then it can be deduced that A not only has the connection between B and C, but also the mapping whose image is message B and message C.

Principle 3: $\frac{A∋B,A|\equiv \#\left(B\right)}{A|\equiv \#\left\{H\left(B\right)\right\}}$

Principle 3 indicates: If A not only has message B, but also trusts that B is the latest message, then, it can be deduced that A also trusts that the equation containing B is the latest.

Principle 4: $\frac{A|\nabla \left(B\right)}{A∋B}$

Principle 4 indicates: If A received message B, then A has message B can be deduced.

Principle 5: $\frac{A|\equiv D\overset{k}{↔}A,\;A\left|\nabla H\left(B,\langle K\rangle \right)\;,\;A∋\left(B,K\right),\;A\right|\equiv \#\left(B,K\right)}{A\left|\equiv D\right|\sim \left(B,\langle K\rangle \right),A\left|\equiv D\right|\sim H\left(B,\langle K\rangle \right)}$

Principle 5 indicates: A trusts that the secret key K is shared by A and D, and A previously received encrypted B by secret key K. A has the connection between message B and secret key K. A trusts that the connection between message B and secret key K is the latest, then, A trusts that D previously sent the connection between message B and the secret key K, and A also trusts that D used to send encrypted B by K.

### 4.3. Protocol Process

Protocol (1): R→T: request

Protocol (2): T→R: FID

Protocol (3): R→T:$\mathrm{FID}\;⊕{\;P}_{n+1}\;⊕{\;K}_{n}\;⊕{\;r}_{1}⊕{\;r}_{2}||\left(\left(r_{1}<\;<\;<8\right)\;\&\;\left(r_{2}<\;<\;<1\right)\;⊕\left({\;P}_{n+1}<\;<\;<2\right)\right)$

Protocol (4): T→R:$\left(\mathrm{PUF}\left(P_{n}\right)\;⊕\;\mathrm{PUF}\left(\mathrm{PUF}\left(P_{n}\right)\right)⊕{\;r}_{2}^{\prime },\;\left(\mathrm{FID}<\;<\;<\;8\right)\;\&\;\left(\;\mathrm{PUF}\left(P_{n}\right)\;⊕\;\mathrm{PUF}\left(\mathrm{PUF}\left(P_{n}\right)\right)⊕{\;r}_{2}^{\prime }<\;<\;<1\right)\;⊕\;\left(r_{2}^{\prime }<\;<\;<2\right)\right)$

Protocol (5): R→T: success/failure

### 4.4. Specifically Describe the Protocol above by Using the GNY Logic Language

Protocol (1): T|∇ request

Protocol (2): R|∇ FID

Protocol (3): $T\left|\nabla \mathrm{FID}\;⊕{\;P}_{n+1}\;⊕{\;K}_{n}\;⊕{\;r}_{1}⊕{\;r}_{2}\right||\left(\left(r_{1}<\;<\;<8\right)\;\&\;\left(r_{2}<\;<\;<1\right)\;⊕\;\left({\;P}_{n+1}<\;<\;<2\right)\right)$

Protocol (4): R|∇$\left(\mathrm{PUF}\left(P_{n}\right)\;⊕\;\mathrm{PUF}\left(\mathrm{PUF}\left(P_{n}\right)\right)⊕{\;r}_{2}^{\prime },\;\left(\mathrm{FID}<\;<\;<\;8\right)\;\&\;\left(\;\mathrm{PUF}\left(P_{n}\right)\;⊕\;\mathrm{PUF}\left(\mathrm{PUF}\left(P_{n}\right)\right)⊕\;r\;\prime <\;<\;<1\right)\;⊕\;\left(r_{2}^{\prime }<\;<\;<2\right)\right)$

Protocol (5): T|∇ success/failure

### 4.5. Assumption

(1) $\;T∋\left(\mathrm{FID},{\;P}_{n},{\;K}_{n},\;\mathrm{ID}\right)$
(2) $\;R∋\left({\mathrm{FID}}^{\mathrm{new}},{\;P}_{n}^{\mathrm{new}},{\;P}_{n+1}^{\mathrm{new}},{\;K}_{n}^{\mathrm{new}},{\;\mathrm{FID}}^{\mathrm{old}},{\;P}_{n}^{\mathrm{old}},{\;P}_{n+1}^{\mathrm{old}},\;\mathrm{ID},{\;K}_{n}^{\mathrm{old}}\right)$
(3) $\;T∋\left(r_{1}\right)$
(4) $\;T|\equiv \#\left(r_{1}\right)$
(5) $\;R∋\left(r_{2}\right)$
(6) $\;R|\equiv \#\left(r_{2}\right)$
(7) $\;T|\equiv R\overset{P_{n},{\;K}_{n},{\;P}_{n+1}}{↔}T$
(8) $\;R|\equiv T\overset{P_{n},{\;K}_{n},{\;P}_{n+1}}{↔}R$

### 4.6. Target Formulas to Be Proven

(1) $\;T\left|\equiv R\right|\sim \#\left\{\mathrm{FID}\;⊕{\;P}_{n+1}\;⊕{\;K}_{n}\;⊕{\;r}_{1}⊕{\;r}_{2},\;\left(r_{1}<\;<\;<8\right)\;\&\;\left(r_{2}<\;<\;<1\right)\;⊕\;\left({\;P}_{n+1}<\;<\;<2\right)\right\}$
(2) $\;R\left|\equiv T\right|\sim \#\left\{\mathrm{PUF}\left(P_{n}\right)\;⊕\;\mathrm{PUF}\left(\mathrm{PUF}\left(P_{n}\right)\right)⊕{\;r}_{2}^{\prime },\;\left(\mathrm{FID}<\;<\;<8\right)\;\&\;\left(\mathrm{PUF}\left(P_{n}\right)\;⊕\;\mathrm{PUF}\left(\mathrm{PUF}\left(P_{n}\right)\right)⊕{\;r}_{2}^{\prime }<\;<\;<1\right)\;⊕\;\left(r_{2}^{\prime }<\;<\;<2\right)\right\}$

(1) Prove $T\left|\equiv R\right|\sim \#\left\{\mathrm{FID}\;⊕{\;P}_{n+1}\;⊕{\;K}_{n}\;⊕{\;r}_{1}⊕{\;r}_{2},\;\left(r_{1}<\;<\;<8\right)\;\&\;\left(r_{2}<\;<\;<1\right)\;⊕\;\left({\;P}_{n+1}<\;<\;<2\right)\right\}$

S1: by principle: $\frac{A|\equiv \#\left(B\right)}{A\left|\equiv \#\left(B,C\right),A\right|\equiv \#\left(F\left(B\right)\right)}$ and assumption (4)$\;T|\equiv \#\left(r_{1}\right)$, we can deduce:

$$T|\equiv \#\left\{\mathrm{FID}\;⊕{\;P}_{n+1}\;⊕{\;K}_{n}⊕{\;r}_{1}⊕{\;r}_{2}\right\}$$

$$T|\equiv \#\left\{\left(r_{1}\right)\;\&\;\left(r_{2}\right)\;⊕\;\left({\;P}_{n+1}\right)\right\}$$

S2: by principle: $\frac{A∋B,A∋C}{A∋\left(B,C\right),A∋\left(F\left(B,C\right)\right)}$ and assumption (3)$\;T∋\left(r_{1}\right)$, we can deduce:

$$T∋\left\{\left(r_{1}\right)\;\&\;\left(r_{2}\right)\;⊕\;\left({\;P}_{n+1}\right)\right\}$$

S3: by principle: $\frac{A∋B,A|\equiv \#\left(B\right)}{A|\equiv \#\left\{H\left(B\right)\right\}}$ and the above corollary, we can deduce:

$$T|\equiv \#\left\{\left(r_{1}<\;<\;<8\right)\;\&\;\left(r_{2}<\;<\;<1\right)\;⊕\;\left({\;P}_{n+1}<\;<\;<2\right)\right\}$$

S4: by principle: $\frac{A|\nabla \left(B\right)}{A∋B}$ and protocol (3), we can deduce:

$$T∋\left\{\mathrm{FID}\;⊕{\;P}_{n+1}\;⊕{\;K}_{n}\;⊕{\;r}_{1}⊕{\;r}_{2}\right\}$$

$$T∋\left\{\left(r_{1}<\;<\;<8\right)\;\&\;\left(r_{2}<\;<\;<1\right)\;⊕\;\left({\;P}_{n+1}<\;<\;<2\right)\right\}$$

S5: by principle: $\frac{A|\equiv D\overset{k}{↔}A,\;A\left|\nabla H\left(B,\langle K\rangle \right)\;,\;A∋\left(B,K\right),\;A\right|\equiv \#\left(B,K\right)}{A\left|\equiv D\right|\sim \left(B,\langle K\rangle \right),A\left|\equiv D\right|\sim H\left(B,\langle K\rangle \right)}$ and assumption (7) and (8), we can deduce:

$$T\left|\equiv R\right|\sim \left\{\mathrm{FID}\;⊕{\;P}_{n+1}\;⊕{\;K}_{n}\;⊕{\;r}_{1}⊕{\;r}_{2}\right\}$$

$$T\left|\equiv R\right|\sim \left\{\left(r_{1}<\;<\;<8\right)\;\&\;\left(r_{2}<\;<\;<1\right)\;⊕\;\left({\;P}_{n+1}<\;<\;<2\right)\right\}$$

S6: then, there is:

$$T\left|\equiv R\right|\sim \#\left\{\mathrm{FID}\;⊕{\;P}_{n+1}\;⊕{\;K}_{n}\;⊕{\;r}_{1}⊕{\;r}_{2}\right\}$$

$$T\left|\equiv R\right|\sim \#\left\{\left(r_{1}<\;<\;<8\right)\;\&\;\left(r_{2}<\;<\;<1\right)\;⊕\;\left({\;P}_{n+1}<\;<\;<2\right)\right\}$$

S7: by the definition, we can also deduce:

$$T\left|\equiv R\right|\sim \#\left\{\mathrm{FID}\;⊕{\;P}_{n+1}\;⊕{\;K}_{n}\;⊕{\;r}_{1}⊕{\;r}_{2},\;\left(r_{1}<\;<\;<8\right)\;\&\;\left(r_{2}<\;<\;<1\right)\;⊕\;\left({\;P}_{n+1}<\;<\;<2\right)\right\}$$

(2) prove

$R\left|\equiv T\right|\sim \#\left\{\mathrm{PUF}\left(P_{n}\right)\;⊕\;\mathrm{PUF}\left(\mathrm{PUF}\left(P_{n}\right)\right)⊕{\;r}_{2}^{\prime },\;\left(\mathrm{FID}<\;<\;<8\right)\;\&\;\left(\mathrm{PUF}\left(P_{n}\right)\;⊕\;\mathrm{PUF}\left(\mathrm{PUF}\left(P_{n}\right)\right)⊕{\;r}_{2}^{\prime }<\;<\;<1\right)\;⊕\;\left(r_{2}^{\prime }<\;<\;<2\right)\right\}$

S1: by principle: $\frac{A|\equiv \#\left(B\right)}{A\left|\equiv \#\left(B,C\right),A\right|\equiv \#\left(F\left(B\right)\right)}$ and assumption (6)$\;R|\equiv \#\left(r_{2}\right)$, there is:

$$R|\equiv \#\left\{\mathrm{PUF}\left(P_{n}\right)\;⊕\;\mathrm{PUF}\left(\mathrm{PUF}\left(P_{n}\right)\right)⊕{\;r}_{2}^{\prime }\right\}$$

$$R|\equiv \#\left\{\left(\mathrm{FID}\right)\;\&\;\left(\mathrm{PUF}\left(P_{n}\right)\;⊕\;\mathrm{PUF}\left(\mathrm{PUF}\left(P_{n}\right)\right)⊕{\;r}_{2}^{\prime }\right)\;⊕\;\left(r_{2}^{\prime }\right)\right\}$$

S2: by principle: $\frac{A∋B,A∋C}{A\left|∋\left(B,C\right),A\right|∋\left(F\left(B,C\right)\right)}$ and assumption (5)$\;R∋\left(r_{2}\right)$, there is:

$$R∋\left\{\left(\mathrm{FID}\right)\;\&\;\left(\mathrm{PUF}\left(P_{n}\right)\;⊕\;\mathrm{PUF}\left(\mathrm{PUF}\left(P_{n}\right)\right)⊕{\;r}_{2}^{\prime }\right)\;⊕\;\left(r_{2}^{\prime }\right)\right\}$$

S3: by principle: $\frac{A∋B,A|\equiv \#\left(B\right)}{A|\equiv \#\left\{H\left(B\right)\right\}}$ and the corollary above, there is:

$$R|\equiv \#\left\{\left(\mathrm{FID}<\;<\;<8\right)\;\&\;\left(\mathrm{PUF}\left(P_{n}\right)\;⊕\;\mathrm{PUF}\left(\mathrm{PUF}\left(P_{n}\right)\right)⊕{\;r}_{2}^{\prime }<\;<\;<1\right)\;⊕\;\left(r_{2}^{\prime }<\;<\;<2\right)\right\}$$

S4: by principle: $\frac{A|\nabla \left(B\right)}{A∋B}$ and protocol (4), there is:

$$R∋\left\{\mathrm{PUF}\left(P_{n}\right)\;⊕\;\mathrm{PUF}\left(\mathrm{PUF}\left(P_{n}\right)\right)⊕{\;r}_{2}^{\prime }\right\}$$

$$R∋\left\{\left(\mathrm{FID}<\;<\;<8\right)\;\&\;\left(\mathrm{PUF}\left(P_{n}\right)\;⊕\;\mathrm{PUF}\left(\mathrm{PUF}\left(P_{n}\right)\right)⊕{\;r}_{2}^{\prime }<\;<\;<1\right)\;⊕\;\left(r_{2}^{\prime }<\;<\;<2\right)\right\}$$

S5: by principle: $\frac{A|\equiv D\overset{k}{↔}A,\;A\left|\nabla H\left(B,\langle K\rangle \right)\;,\;A∋\left(B,K\right),\;A\right|\equiv \#\left(B,K\right)}{A\left|\equiv D\right|\sim \left(B,\langle K\rangle \right),A\left|\equiv D\right|\sim H\left(B,\langle K\rangle \right)}$ and assumption (7) and (8), we can deduce:

$$R\left|\equiv T\right|\sim \left\{\mathrm{PUF}\left(P_{n}\right)\;⊕\;\mathrm{PUF}\left(\mathrm{PUF}\left(P_{n}\right)\right)⊕{\;r}_{2}^{\prime }\right\}$$

$$R\left|\equiv T\right|\sim \left\{\left(\mathrm{FID}<\;<\;<8\right)\;\&\;\left(\mathrm{PUF}\left(P_{n}\right)\;⊕\;\mathrm{PUF}\left(\mathrm{PUF}\left(P_{n}\right)\right)⊕{\;r}_{2}^{\prime }<\;<\;<1\right)\;⊕\;\left(r_{2}^{\prime }<\;<\;<2\right)\right\}$$

S6: according to the definition, we can deduce:

$$R\left|\equiv T\right|\sim \#\left\{\mathrm{PUF}\left(P_{n}\right)\;⊕\;\mathrm{PUF}\left(\mathrm{PUF}\left(P_{n}\right)\right)⊕{\;r}_{2}^{\prime }\right\}$$

$$R\left|\equiv T\right|\sim \#\left\{\left(\mathrm{FID}<\;<\;<8\right)\;\&\;\left(\mathrm{PUF}\left(P_{n}\right)\;⊕\;\mathrm{PUF}\left(\mathrm{PUF}\left(P_{n}\right)\right)⊕{\;r}_{2}^{\prime }<\;<\;<1\right)\;⊕\;\left(r_{2}^{\prime }<\;<\;<2\right)\right\}$$

S7: by definition, there is:

$$R\left|\equiv T\right|\sim \#\left\{\mathrm{PUF}\left(P_{n}\right)\;⊕\;\mathrm{PUF}\left(\mathrm{PUF}\left(P_{n}\right)\right)⊕{\;r}_{2}^{\prime },\;\left(\mathrm{FID}<\;<\;<8\right)\;\&\;\left(\mathrm{PUF}\left(P_{n}\right)\;⊕\;\mathrm{PUF}\left(\mathrm{PUF}\left(P_{n}\right)\right)⊕{\;r}_{2}^{\prime }<\;<\;<1\right)\;⊕\;\left(r_{2}^{\prime }<\;<\;<2\right)\right\}$$

## 5. Security Analysis

This section analyses the possible attacks and the security of this protocol.

### 5.1. Mutual Verification

Mutual verification: Reader verifies the tag, and tag reversely verifies the reader. For some verification protocols, they just verify the tag, without the verification of the reader, which results in a security issue. If attacker uses a reader which is not been verified by a tag, the RFID system data would be leaked, or even the system would be damaged irreparably, such as desynchronization attack and DDos attack.

(1) Reader forgery
  The third step of the proposed protocol is to send message A||B to RFID tag, according to the Equations (3) and (4), tag calculates the values of D and E, and tag deduces the random number $r_{2}^{\prime }$ of the reader with Equation (5), where deduced $r_{2}^{\prime }$ is also used to generate  B ′ by Equation (6). Then,  B ′and B of the reader are compared to verify the reader, in which value of A and value of B are calculated by the random number $r_{1}$ of the tag, random number $r_{2}$ of the reader, FID of the tag and the shared secret key $P_{n+1}$ and secret key $K_{n}.\;$These values would be updated after each round to prepare for the next round, and this is because the original values should be secreted so that the attacker cannot produce a fake reader.
(2) Tag forgery
  The fourth step of our protocol is to send message F||H to the reader, reader deduces the secret key of the tag  E ′ by Equation (9), and uses deduced secret key  E ′, its random number $r_{2}$, secret key $P_{n+1}$ and Equation (10) to calculate  F ′. Then reader uses FID of the tag,  F ′, and random number $r_{2}^{\prime }$ to produce  H ′ by Equation (9), by which reader compares H and the calculated  H ′ to complete the verification of the reader. In this process, F and H are calculated by the deduced random number $r_{2}$ by the tag, the secret key produced by PUF and the tag signature FID. After each round verification, these values would be updated for the following verification. As long as the initial values are in secret, attacker cannot produce a fake tag.

Above all, this protocol can guarantee the mutual verification between reader and tag.

### 5.2. Data Confidentiality and Tag Anonymity

During the verification process, the internal data of either tag or reader should be transferred in cipher text, and the data about tag identity should also be cipher text in transition. Although attacker has intercepted some data of the tag or the reader, attacker is not allowed to deduce any message about the identity of reader and tag.

The critical steps of our protocol are the second, third and fourth step. In the second step, tag sends its FID to the reader, which is just alias and has no relation with the real identity of the tag, and it would be updated after each verification round. Although attacker intercepts this alias, it cannot obtain any effective identity message of the tag. The third and the fourth steps send the A||B of the reader to the tag, and send F||H of the tag to the reader respectively. The previous mutual verification has analyzed the random number $r_{1}$ of the tag, random number $r_{2}$ of the reader, FID of the tag and the shared secret key $P_{n+1}$ and secret key $K_{n}$ obtained by calculation. As long as the initialization is in secret, attacker cannot calculate those values. Although attacker can intercept them, those intercepted values cannot be used to deduce the secret key and deduce the tag identity.

Above all, this protocol guarantees the data confidentiality and the integrity of the whole RFID system.

### 5.3. Steal Attack

Attacker steals the data of the reader and the tag to make the data cannot be received by either tag or reader, as a way to damage the RFID system.

Assume that attacker steals the last message (failure/success) from reader to the tag, and promotes reader to update the secret key of the database except for the secret key of the tag. In this way, the data of tag and the database are not synchronous. Our protocol solves this problem by maintaining the FID and secret key $P_{n}$ and $K_{n}$ of the last verification. Even the tag cannot update its secret key value, the database can find its FID.

In summary, this protocol can defend against steal attack.

### 5.4. Replay Attack

Attacker iteratively sends the message that has been received by the reader to cheat reader, which would make reader to response so that the RFID system is damaged.

Assume the attacker intercepts the message during a mutual verification: ①$\mathrm{FID},{\;r}_{1}$; ②A||B; ③F||H. Then, attacker intercepts the last message from reader to the tag (failure/success), and the reader updates the secret value of the database as a result, except for the secret key of tag. In the next verification round, attacker firstly sends $\mathrm{FID},{\;r}_{1}$ that are intercepted previously to the reader. After receiving those message, reader searches this FID and finds that it matches to a ${\mathrm{FID}}^{\mathrm{old}}\;$. Later, reader generates the random number $r_{2}$ and produce A||B by equation. If attacker can send the previously obtained F||H to activate a forgery verification, however, during this verification, the random number generated by the reader is different from the random number produced in the last round, which results in the difference between H and  H ′, so that the tag verification will fail. Above all, this protocol defends against replay attack.

### 5.5. Backtracking Attack

When a RFID tag is deciphered, attacker cannot deduce previous verification information by this current verification.

Assume that an attacker cracks the current verification process, and obtains the FID of the tag and the secret value $(P_{n},{\;K}_{n}$). However, attacker cannot deduce previous secret key values in verification because these secret key values are updated with the random number $r_{1},r_{2}$ after each verification round.

Therefore, this protocol guarantees the safety against the backtracking.

### 5.6. Clone Attack

Attacker copies the legal tag to obtain a clone tag, which contains all information of the copied tag, including the unique ID, data and algorithms stored in tag. In other words, when reader sends a random number and a request to the tag, clone tag can response to the reader in a same way as the legal tag. Thus, simple encryption algorithms and increase of the complexity of the verification algorithms cannot prevent against clone attack.

Our protocol is based on PUF technique to achieve the mutual verification. In the previous introduction of the PUF technique, we have analyzed its safety. Although attacker launches attack on reader or tag, and obtains the secret key of the tag: {$P_{n},\;\mathrm{FID},{\;K}_{n}$}, attacker cannot produce a clone tag with these values. This is because PUF technique is by deriving the difference among chips during manufacture, which causes the different outputs. In this way, clone attack can be prevented against by our protocol.

### 5.7. Desynchronization Attack

Attacker triggers the update of tag by some means except for the update of the reader or vice-versa. As a result, the desynchronization between the data of the database and the tag data exists, and tag cannot be verified by the reader in following verification.

Attack one: Modifying message A or message B

Assume attacker is intercepting a verification process, until the reader sending A||B to the tag, attacker modifies this message into $A^{“}||B^{”}$, and then sends it the tag. After receiving $A^{“}||B^{”}$, tag firstly deduces the random number $r_{2}\;$of the reader by $A^{”}$, by which tag calculates  B ′. Then, tag judges if  B ′ = $B^{”}$ as a way to verify reader. Because attacker modifies message B into $B^{”}$, reader cannot be successfully verified, which defends against the desynchronization caused by modifying message A or message B.

Attack two: Modifying message F or message H.

Assume attacker is intercepting a verification process, until confirmation of the identity of the reader, tag calculates message F||H and sends it to reader to verify. At this moment, attacker modifies this message into $F^{“}||H^{”}$, and sends the modified message to reader. After receiving $F^{“}||H^{”}$, reader firstly deduces the secret key  E ′ by $F^{”},$ then reader calculates  H ′. After this, reader judges if  H ′ = $H^{”}\;$or not to verify the tag. Because attacker modifies message H into $H^{”}$, reader cannot verify tag successfully, so that both tag and reader cannot update their own data, which thus solves the problem of desynchronization caused by modifying F or modifying H.

Compared with previous Kulseng’s lightweight mutual verification protocol, Table 2 shows the advantages of our protocol.

## 6. Experiments

This section shows the secure tag verification in a RFID system according to our protocol. We simulate to construct a vehicle cargo invading detection system, and all vehicles of this system carry RFID tags. With ultrahigh frequency RFID reader, the tag of a vehicle would be recorded, which information is recorded by the database and operating platform executes the secure verification via our protocol.

Ultrahigh frequency reader: Impinj R420. Air interface protocol: EPC global UHF Class 1 Gen 2/ISO 18000-6C, using Autopilot technique of the Speedway Revolution, which is able to automatically optimize the performance of the reader in any environment to maintain the best performance. Autopilot technique has following features:

1. Automatically setting—Optimize the deployment of reader to achieve the best performance.
2. Low load circulation—Reduce radio-frequency interference, power dissipation, energy cost.
3. Dynamic antennae switch—Increase handling capacity, enhance the reader to more effective work.

Speedway Revolution RFID reader also supports power over Ethernet (PoE), increases the flexibility of its application, so that the deployment is simplified and does not need AC power supply. As a result, the cost is dramatically reduced. Reader is equipped with the capability of the best flexibility in receiving, anti-interference and product and carrier offset capability.

RFID tag: Impinj H47, which supports air interface protocol EPC Class 1 Gen 2/ISO18000-6C; working frequency: 860~960 MHz, read distance is decided by the reader emit power. The tag can be fixed on the object in a sticker manner, and is easy to be read for obtaining information.

Platform construction tools: Java development platform.

Operating system: Windows 10.

In this section, we mainly discuss the RFID readers of what frequency we choose. Different frequency readers are limited by different distance when readers read tags’ information. If the distance is too long, the reader cannot read the information of the tag, which will lead to the occurrence of missed reading. The counterfeiters can mix the real and fake tags together and put the fake tags out of the range to allow them to cheat the reader. Therefore, it is important to discuss how to use this protocol in terms of different frequencies and ranges of RFID readers.

**Figure 6:**
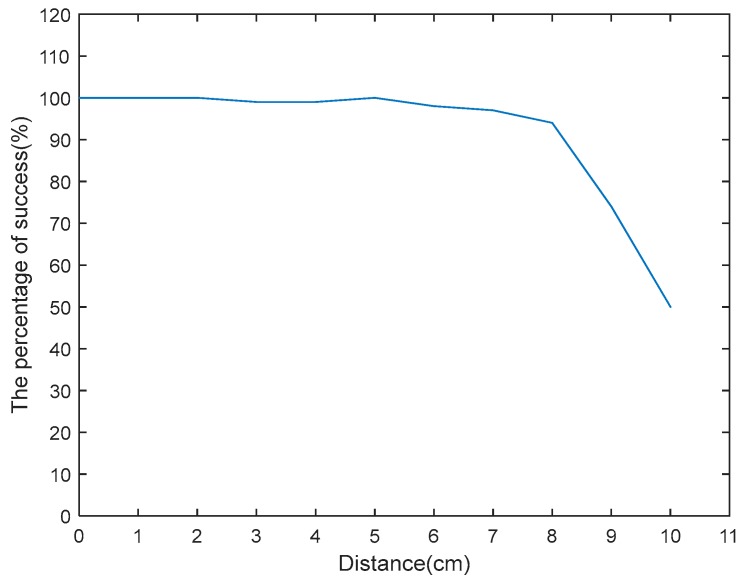
High frequency experiments.

In the experiments, to verify the impact of using high frequency reader and the ultrahigh frequency reader on the system alarm’s success rate, in the cases with different distance between the tag and the reader, through experiencing 100 times experimental result analysis, the results are shown by Figure 6, we can conclude: within 10 cm from high frequency RFID reader, the number of successfully finding the clone tag are: 100, 100, 100, 99, 99, 100, 98, 97, 94. The distance between the reader and tag is over the best reading distance 8 cm, because the Mifare card data cannot be effectively read and the success rate dramatically decreases.

**Figure 7:**
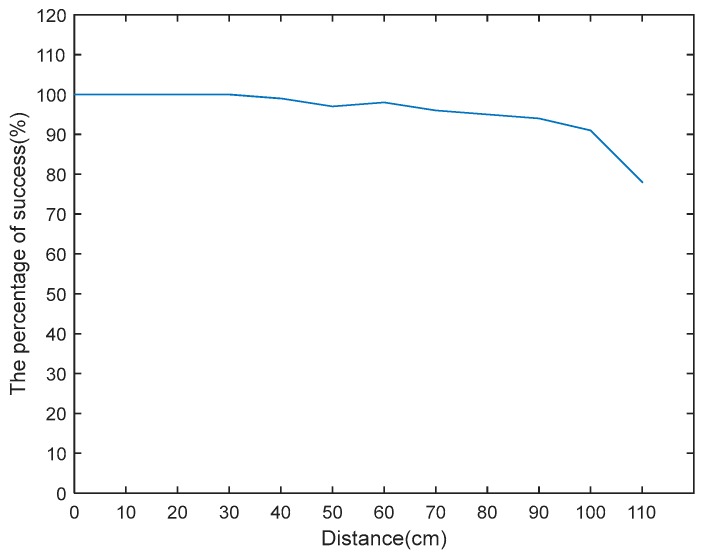
Ultra-high frequency experiments.

As shown in Figure 7, in conclusion: within 110 cm from the RFID ultrahigh frequency reader, times of the reader being attacked are respectively: 100, 100, 100, 100, 99, 97, 98, 96, 95, 94, 91, 78. When the effective distance is bigger than the nominal range 100 cm, because reader cannot obtain the tag data, success rate obviously reduces. Experimental sample data is given in Figure 7.

This experiment indicates when the high frequency reader is located over the nominal reading range, which is more than 8 cm, the performance dramatically falls, the rate of invading tag dramatically reduces. With our experimental sample, comprehensive success rate is 91.90%; while the ultrahigh frequency reader’s performance slightly reduces when the distance is over the nominal reading distance, when the distance is over 90 cm, the performance of the reader begins to reduce. In comparison, ultrahigh frequency RFID reader of the original system performs better in detecting fake tag.

## 7. Performance Analysis

By calculating the costs of time and storage space for a complete certification process of backstage database, reader and tag are used to judge the performance of the proposed protocol. In this section, the reader and the backend database are viewed as a whole for the convenience of analysis, which is called the reader side. For the protocol presented in this paper, the data stored in the tag, reader, and back-end database is a unit of 96 bits, let L substitute for 96 bits. $T_{P}$ defines as the number of PUF function operations. $T_{R}\;$defines as the number of times when a random number is generated. $T_{\mathrm{XOR}}$ defines as the number of XOR operation.${\;T}_{\mathrm{LEFT}}\;$defines as the number of times of the loop left movement, $T_{\mathrm{OR}}$ defines as the number of join operation, $T_{\mathrm{AND}}$ defines as the number of AND operation, and $T_{F}$ defines as the number of F function operation. Then the complete certification process will be analyzed in the term of its complexity.

Firstly, we analyze the time complexity.

For the RFID tag, in tag verification phase, a random number of $r_{1}$ is generated in the tag recognition phase, so the time complexity of the tag is $T_{R}$; in the process of authenticating the reader of Mutual Verification phase, there are four operations by a tag, such as $D=\mathrm{PUF}\left(P_{n}\right),\;E=\mathrm{PUF}\left(D\right),{\;r}_{2}^{\prime }=A\;⊕\;\mathrm{FID}\;⊕\;D\;⊕{\;K}_{n}⊕{\;r}_{1},\;B\;\prime =\left(r_{1}<\;<\;<8\right)\;\&\;\left(r_{2}^{\prime }<\;<\;<1\right)\;⊕\;\left(D<\;<\;<2\right)$. So in the process of authenticating the reader of Mutual Verification phase, the time complexity of the tag is $(2T_{P}+5T_{\mathrm{XOR}}+3T_{\mathrm{LEFT}}+T_{\mathrm{AND}}$). In the process of authenticating the tag of Mutual Verification phase, there are three operations by a tag, such as$\;F=D\;⊕\;E⊕{\;r}_{2}^{\prime },\;H=\left(\mathrm{FID}<\;<\;<8\right)\;\&\;\left(F<\;<\;<1\right)\;⊕\;\left(r_{2}^{\prime }<\;<\;<2\right)$and F||H. So in the process of authenticating the tag of Mutual Verification phase, the time complexity of the tag is $(3T_{\mathrm{XOR}}+3T_{\mathrm{LEFT}}+T_{\mathrm{AND}}+T_{\mathrm{OR}}$). In the whole Mutual Verification phase, the time complexity of the tag is $(2T_{P}+8T_{\mathrm{XOR}}+6T_{\mathrm{LEFT}}+2T_{\mathrm{AND}}+T_{\mathrm{OR}}$). In the update phase, there are two operations by a tag, such as $\mathrm{FID}=\left(\mathrm{FID}<\;<\;<8\right)\;\&\;\left(r_{2}^{\prime }<\;<\;<1\right)\;⊕\;\left(r_{1}<\;<\;<2\right)\;$and $K_{n}=\left(K_{n}<\;<\;<8\right)\;\&\;\left(r_{2}^{\prime }<\;<\;<1\right)\;⊕\;\left(r_{1}<\;<\;<2\right)$. Thus, the time complexity of the tag is $(2T_{\mathrm{XOR}}+6T_{\mathrm{LEFT}}+2T_{\mathrm{AND}}$) in the update phase.

For the reader side, in the process of authenticating the reader of Mutual Verification phase, there are three operations by the reader side, such as $A=\mathrm{FID}\;⊕{\;P}_{n+1}⊕{\;K}_{n}\;⊕{\;r}_{1}⊕{\;r}_{2},\;B=\left(r_{1}<\;<\;<8\right)\;\&\;\left({\;r}_{2}<\;<\;<1\right)\;⊕\;\left({\;P}_{n+1}<\;<\;<2\right)$ and A||B. So in the process of authenticating the reader of Mutual Verification phase, the time complexity of the reader side is $(T_{R}+5T_{\mathrm{XOR}}+3T_{\mathrm{LEFT}}+T_{\mathrm{AND}}+T_{\mathrm{OR}}$). In the process of authenticating the tag of Mutual Verification phase, there are three operations by the reader side, such as $\;E\;\prime =F\;⊕{\;r}_{2}\;⊕{\;P}_{n+1},\;F\;\prime =\;E\;\prime ⊕{\;r}_{2}\;⊕{\;P}_{n+1},\;H\;\prime =\left(\mathrm{FID}<\;<\;<8\right)\;\&\;\left(\;F\;\prime <\;<\;<1\right)\;⊕\;\left(r_{2}<\;<\;<2\right)$. So in the process of authenticating the tag of Mutual Verification phase, the time complexity of the reader side is $(5T_{\mathrm{XOR}}+3T_{\mathrm{LEFT}}+T_{\mathrm{AND}}$). So in the whole Mutual Verification phase, the time complexity of the reader side is $(T_{R}+10T_{\mathrm{XOR}}+6T_{\mathrm{LEFT}}+2T_{\mathrm{AND}}+T_{\mathrm{OR}}$). In the update phase, there are two operations by the reader side, such as${\;K}_{n}^{\mathrm{new}}=\left(K_{n}^{\mathrm{new}}<\;<\;<8\right)\;\&\;\left(r_{2}^{\prime }<\;<\;<1\right)\;⊕\;\left(r_{1}<\;<\;<2\right)$ and ${\mathrm{FID}}^{\mathrm{new}}=\left({\mathrm{FID}}^{\mathrm{new}}<\;<\;<8\right)\;\&\;\left(r_{2}^{\prime }<\;<\;<1\right)\;⊕\;\left(r_{1}<\;<\;<2\right)$. So in the update phase, the time complexity of the reader side is $(2T_{\mathrm{XOR}}+6T_{\mathrm{LEFT}}+2T_{\mathrm{AND}}$). Table 3 and Table 4 give the results of the time cost analysis of our protocol and the K protocol.

Secondly, we analyze the space complexity.

For the RFID tag, there are some data in a tag, such as $P_{n},\;\mathrm{FID},{\;K}_{n}$. Thus, the space complexity of a tag is 3L.

For the reader side, there are some data in the reader side, such as ${\mathrm{FID}}^{\mathrm{old}},{\;P}_{n}^{\mathrm{old}},{\;P}_{n+1}^{\mathrm{old}},\;\mathrm{ID},{\;K}_{n}^{\mathrm{old}},{\;\mathrm{FID}}^{\mathrm{new}},{\;P}_{n}^{\mathrm{new}},{\;P}_{n+1}^{\mathrm{new}},{\;K}_{n}^{\mathrm{new}}$. So the space complexity of the reader side is 9 L. The comparison results are shown in the Table 5.

In our protocol, it only uses some simple operations, such as XOR operation, the loop left movement operation, JOIN operation, and AND operation. In addition to PUF circuit by the arbiter judged by two signal circuit who arrives firstly, the amount of calculation is very small. So our protocol meets the requirements of low cost in computational overhead. To sum up, although the protocol proposed in this paper is more complex than the K protocol, its security is much higher than that of K protocol. Therefore, this protocol has found a good balance between performance and security.

## 8. Conclusions

In this paper, a lightweight RFID security protocol based on the Physical Unclonable Function (PUF) is proposed to achieve efficient verification of a single tag. The protocol includes three process: Tag recognition, mutual verification and update. The tag recognition is that the reader recognizes the tag; mutual verification is that the reader and tag mutually verify the authenticity of each other; update is supposed to maintain the latest secret key for the following verification. The results of security show that the presented protocol is efficient to protect RFID systems. Implementation of the presented protocol in real RFID systems are our future work.

## Figures and Tables

**Table 1:** The definitions of K protocol.

Symbol	Definition
ID	The ID of tag
FID	The false ID of tag
IDS	The index of tag in the database
PUF(X)	The result of the value X processed by the PUF module in the tag
⨁	The XOR operation
F(X)	The Permutation function
$K_{n}$	The shared secret key by the reader and the tag
$P_{n}$	The secret value of the tag
< < <	The loop left movement operation
&	The AND operation
||	The JOIN operation

**Table 2:** Comparison of security analysis between the proposed protocol and Kulseng’s lightweight mutual verification protocol.

Types of Attack	Proposed Protocol	K Protocol
Mutual verification	√	√
Data confidentiality and Tag anonymity	√	×
Steal attack	√	×
Replay attack	√	√
Backtracking attack	√	×
Clone attack	√	√
Desynchronization attack	√	×

**Table 3:** Results of the time cost analysis of K protocol.

Device	Tag Verification Phase	Mutual Verification Phase	Update Phase
Tag	-	$2T_{P}+3T_{XOR}+2T_{F}$	$T_{XOR}+T_{F}$
Reader	-	$2T_{XOR}+2T_{F}$	$T_{XOR}+T_{F}$

**Table 4:** Results of the time cost analysis of the proposed protocol.

Device	Tag Verification Phase	Mutual Verification Phase	Update Phase
Tag	$T_{R}$	$2T_{P}+8T_{XOR}+6T_{LEFT}+2T_{AND}+T_{OR}$	$2T_{XOR}+6T_{LEFT}+2T_{AND}$
Reader	-	$T_{R}+10T_{XOR}+6T_{LEFT}+2T_{AND}+T_{OR}$	$2T_{XOR}+6T_{LEFT}+2T_{AND}$

**Table 5:** Results of space cost analysis between the proposed protocol and K protocol.

Protocol	Tag	Reader
K protocol	3 L	4 L
Our protocol	3 L	9 L
